# Ultrasonic and microwave treatment improved jujube juice yield

**DOI:** 10.1002/fsn3.1713

**Published:** 2020-07-03

**Authors:** Youwei Yu, Xiaowen Cheng, Chenxing Zhang, Jingru Zhang, Shaoying Zhang, Jianguo Xu

**Affiliations:** ^1^ College of Food Science Shanxi Normal University Linfen China

**Keywords:** dried jujube, juice yield, mechanism, microwave, ultrasonic

## Abstract

The methods to leaching juice from dried jujube using six treatments and some factors related with juice yield such as total soluble solids (TSS), pectinase activity, pectin contents, galacturonic acid, and the microstructure morphology of cell were investigated. Six treatments including natural leaching, ultrasonic, microwave, ultrasonic before microwave, ultrasonic after microwave, and pressing directly were applied to extract juice. The group which treating with ultrasonic before microwave displayed its total soluble solids (TSS) was 16 °Brix, which was 6.67% higher than that of natural leaching. And its total soluble solids (TSS) reached to equilibrium in 2 hr, which was faster than that of natural leaching. The mechanism of improving the production efficiency of juice yield using ultrasonic combined with microwave was explored accordingly. The content of pectin and galacturonic acid increased by 58.52% and 59.01%, respectively, which were the highest among all samples. The activity of pectinase was 9.71 μg/(h·g), which was significantly decreased 40.23% as compared to natural leaching. And the treated cells became shriveled and pitted, which led to the leakage of the contents of cell. Thus, the result showed that treating with ultrasonic before microwave displayed the best juice yield. Ultrasonic cooperate with microwave was an efficient method to leaching juice from dried jujube.

## Graphical abstract

The methods to leaching juice from dried jujube using ultrasonic combined with microwave were investigated. The result showed that treating dried jujube with ultrasonic before microwave displayed the best juice yield. And its TSS reached to equilibrium in 2 hr, which was faster than that of natural leaching, accelerating the ratio of the juice output at the same time. The mechanism of improving the production efficient of juice yield using ultrasonic combined with microwave was explored accordingly.

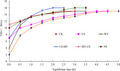

## 1 INTRODUCTION

The effect of sonication treatments on some important quality parameters of juice suggested that sonication technique may successfully be implemented an industrial scale for the processing of juice (Ertugay & Başlar, 2014). Thermosonication (TS) is employed to replace the conventional thermal processing, which can reduce the impact on the nutritional content and overall quality of fruit and vegetable juices (Anaya‐Esparza et al., 2017). Due to the juice industry deteriorated, it is entirely reasonable to focus on the methods for improving the technologies of their production. Ultrasonic treatment is a major potential technique used in the food industry for numerous processes, such as extraction and improving the quality of food (Valero et al., 2007). The substantial action of ultrasound is mainly due to the mechanical property, thermal property, cavitation, and chemical process, in which micro‐bubbles are produced and collapsed within a medium, and then produce cell disruption, the content of cells flowed toward matter's surfaces, or the liquid medium finally (Bermúdez‐Aguirre & Barbosa‐Cánovas, 2012). Cell disruption made extraction and processing more efficient. The ultrasonic device is cheap, simple, and small, and its operation is easy (Chen, Tang, Chen, Wang, & Li, 2010). Compared to other techniques, ultrasound has mild extraction conditions, fast extraction speed, and a wide application range. The advantages are high extract efficiency and short reaction time (Wang, Vanga, & Raghavan, 2019). In recent years, it has attracted widespread attention of researchers and has become a hot topic on food industry. It can be used alone or in combination with other processing technologies.

Microwaves are high‐frequency electromagnetic waves with frequency variation from 300 MHz to 300 GHz (Chandrasekaran, Ramanathan, & Basak, 2013). Microwave has been extensively used to preserve or extract chemical from food products. It can produce alternating magnetic field, make the molecule in the material produce instantaneous polarization, and then do high‐speed thermal movement (Chizoba Ekezie, Sun, Han, & Cheng, 2017). Although the heated food reaches a high temperature quickly, it is worth noting that nonuniform temperature distribution during microwave heating of food materials was in existence. Microwave has gained popularity in food processing due to the characteristics of good penetration, high selectivity, significant reduction in heating time, high efficiency, energy saving, ease and safe of operation, and low maintenance (Kubo, Curet, Augusto, & Boillereaux, 2019). Moreover, owing to contact with food indirectly, it is cleaner and can achieve the selective extraction of substances meanwhile. To some extent, microwave heating might change flavor and nutritional qualities of food more or less as opposed to conventional processing (Benlloch‐Tinoco, Igual, Rodrigo, & Martínez‐Navarrete, 2013).

Jujube (*Ziziphus jujuba Mill*.) is popular for its high nutritional value and medicinal uses (Reche et al., 2018). Jujube fruit provides rich contents of natural bioactive nutrients such as ascorbic acid, amino acids flavonoids, and terpenoids, which are responsible for various pharmacological activities (Siriamornpun, Weerapreeyakul, & Barusrux, 2015). Jujube juice is one of the products originating from jujube processing. Owing to the low moisture content of dried jujube, the juice of dried jujube did not respond well to pressing. To acquiring jujube juice, the whole dried jujube without any destroy was usually leached, or grinded with water to become into pulp. However, the production efficiency of leaching is lower and its juice yield is lower as well. The method to be grinded might quickly acquire juice, but clarification of juice is difficult. Moreover, there was some bitter taste in jujube juice whether leaching or grind method, and consumers often struggle to take it in (Jiang, Li, Wang, Li, & Hong, 2016; Li, Ding, Chen, & Zeng, 2013).

A combination of ultrasound and microwaves widely used in the extraction as innovative “green” food processing technique, and both of them have an important role in promoting sustainable food industry (Su, Zhang, Adhikari, Mujumdar, & Zhang, 2018). However, there is no report on the compound method of the two technologies in the extraction of juice and the synergistic effect of ultrasound and microwave had not been discussed in juice industry. In our experiments, the dried jujubes were firstly sliced, and then were treated with microwave combined with ultrasonic. And the jujube juice yield was researched. Further, the mechanism related with jujube juice was investigated from the point of cell wall such as pectin, galacturonic acid, pectinase, and microstructure. This research might provide an efficient method to acquire juice originating dried fruit, and expand the applications of ultrasonic and microwave in food industry.

## 2 MATERIALS AND METHODS

### 2.1 Materials and reagents

The fresh jujube (*Ziziphus jujuba Mill. cv. junzao*) was picked in mid‐September and fully mature jujube was selected to for drying. The jujubes were picked by hand and the peel of jujube became dark red, slightly wrinkled, and soft at the condition of high maturity. The dried jujubes were purchased from a local market in Yaodu District (Shanxi, China). The jujubes in the experiment possessed some features, such as dark red color, spindle shape, varied in length from 3–5 cm, the percentage of edible part exceeds a certain number (85%), and the percentage of water content was approximately 20%. And they were stored at room temperature, cool, and dry, away from light, heat, ventilation, humidity ≤60%. Then, they were selected for absence of defects and rot.

The main reagents such as pectin, carbazole (purity ≥96.0%), and D‐(+)‐galacturonic acid monohydrate (purity ≥97.0%) were purchased from Sino‐pharm Chemical Reagent Co., Ltd. Alfa Aesar Company supplied other regents. All chemicals were of analytical grade.

### 2.2 Sample treating and jujube juice acquirement

The dried jujubes were washed using tap water to remove the surface blemishes. And then, the date pits were discarded. Afterward, the cleaned jujubes were cut into slices with the thickness of approximately 3 mm. Based on preliminary studies, a bulk solution of the jujube pulp was prepared by mixing the pure pulp with water at a ratio of 25% w/w. Namely, the weight ration of jujube and water is 1:4. The mixture was treated by six methods immediately when the two substances mixed well. The natural leaching group was mixing without any treatment and leaching directly, while the pressing was squeezed by a high‐speed blender for some seconds and then leaching. On the basis of the natural leaching group, it was retreated by ultrasonic and microwave. And the two processes were next to each other in the composite processing of ultrasonic and microwave.

The bulk solutions were differently processed as followed. All the selected conditions were obtained from the preliminary experiments and they could cause the results to the best. There were six different treatment methods, including natural leaching (as control check, CK), ultrasonic (US), microwave (MV), ultrasonic before microwave (US‐MV), microwave before ultrasonic (MV‐US), and pressing (PS), of which the experiment was mentioned. The ultrasonic (JY92‐IIDN, Ningbo, China) power and the ultrasonic time were maintained at 540 W, and 15 min, respectively. The microwave (G70D20CN1P‐D2, Guangdong, China) treatment of mixture was carried out at following condition: the power of 560 W for 2 min. All the treatments were finished at a water‐to‐pulp ratio of 4:1.

After the juice soluble solids of each leached jujube sample no longer changed, the mixture with juice and pomace was filtered. The total soluble solids of the juice were measured by a handheld refractometer (ATAGO). Collecting jujube juice and pulp respectively, and stored in the freezer for further analysis.

### 2.3 Analytical determinations

#### 2.3.1 The total pectin and water‐soluble pectin of jujube juice

Pectin of juice samples was determined using carbazole method developed with some modifications (Ningaé et al., 2018). Pectin was precipitated by adding 25 ml of 95% w/w ethanol at 75°C to a 50 ml calibrated centrifuge tube with 2 ml of juice. The obtained mixture was heated in an 85°C water bath for 10min and stirred thoroughly. 95% w/w ethanol was added to bring the total volume to 50 ml. A gel containing insoluble solids in ethanol was formed and then separated by centrifugation for 10 min at room temperature, and the supernatant was discarded. The mixture was immersed in a water bath (DZKE‐D‐2) at 85°C when the precipitation was washed by 63% w/w ethanol and then centrifuged. To remove the monose, the pectin precipitate was washed thrice with ethanol. The precipitate remaining in the centrifuge tubes was collected and dissolved in 1 M NaOH solution. It was dissolved in distilled water to a volume of 50 ml. The samples were then allowed to stand for 15 min to deesterify the pectin. The total pectin and soluble pectin of jujube juice were determined after filtrating. Each sample was analyzed in triplicate by mixing 0.5 ml sample with 0.25 ml of 0.15% w/v carbazole reagent. While the floccus precipitate generated, 3 ml of H_2_SO_4_ was introduced in test tubes which were held in a water bath at 85°C. The tubes were heated for 5 min, and then allowed to cool at room temperature for 15 min. The mixtures in the tubes were used for colorimetric determination. The transmittance was measured at 530 nm using a spectrophotometer (752N).

#### 2.3.2 The water‐soluble pectin, protopectin, and total pectin of jujube pulp

The pectin was extracted according to the method reported previously by Alvarez, Alvarez, Riera, and Coca (1998) with some modifications. The pulp (2 g) ground in a mortar was suspended in 25 ml 95% w/w ethanol taken, transferred into a 50 ml graduated centrifugal tube, and kept it in a boiling‐bath for 30 min. It is crucial to add 95% w/w ethanol in time during boiling. After cooling at room temperature, centrifuge for 15 min and discard the supernatant. Then repeat the steps above mentioned for 2–3 times.

The precipitation in the original test tube dissolved with distilled water (20 ml), and hydrolysis took place at a constant 50°C for 30 min in a water bath. Centrifugal for 15 min till the tubes were cool. The precipitation washed by distilled water not only once. All the supernatant placed and dissolved into a volume of 50 ml finally. The obtained precipitation by hydrolyzation was dissolved with the solution of 0.5 M sulfuric acid. Heating for 1 hr to dissolve protopectin in a boiling‐bath and cooling, then the precipitate is removed by centrifugation and the supernatant extract is analyzed for analytics. Pectin of samples was determined using carbazole method as was mentioned above. The experiments were performed in triplicate. The content of total pectin is the sum of water‐soluble pectin and protopectin.

#### 2.3.3 Galacturonic acid

The mixture was made by slowly adding 8 ml anhydrous ethanol to 2 ml of juice and precipitated at 4°C overnight, followed by centrifugation at 5,000 r/min, for 10 min. The precipitation was washed twice with anhydrous ethanol, acetone, and ether, respectively. Distilled water was added to dissolve the polysaccharide obtained to 50 ml (Yang, Mu, & Ma, 2019). The pulp (2 g) tested was added to 20 ml distilled water and placed in a 50 ml centrifuge tube. The mixture water bath at 90°C for 1 hr and repeated for 2–3 times and then centrifuged while it is at room temperature. The samples (0.5 ml) with 3 ml of H_2_SO_4_ were held in a water bath at 85°C for 20 min after stirring thoroughly. The test tubes were cooled in an ice bath, and 0.25 ml of 0.15% w/v carbazole reagent was added. The mixture was allowed to stand for 2 hr, and the transmittance was measured at 520 nm using a spectrophotometer.

#### 2.3.4 Pectinase activity

Diluted juice was tested directly. The pulp tissue chopped (2 g) was homogenized with a small amount of quartz sand in a mortar for a period of time with 2 ml of citric acid‐phosphate buffer (pH 7.6). Then washed with buffer solution for 3 times and centrifuged at 5,000 r/min for 15 min at 4°C. The supernatant as a crude enzyme solution was kept at low temperature until used. Two 10 ml colorimetric tubes with 2.5 ml of pectin marked as a and b respectively were prepared to preheat for 5 min at 50°C in a water bath. 2 ml of citric acid‐phosphate buffer (pH 5.0) and 0.5 ml of diluted enzyme liquid were added to the tubes. Tube a response accurately for 30 min in the water bath at 50°C and tube b boiled for 5 min immediately. A reaction mixture of 0.8 ml of the samples from the tubes, 0.8 ml of distilled water, and 2 ml of 3, 5‐dinitrosalicylic acid reagent was stirred adequately. The reagent system was boiled for 5 min, and then immediately cooled with tap water. The rate of reaction in absorbance at 540 nm was recorded (Khurnpoon, Siriphanich, & Labavitch, 2008).

#### 2.3.5 Microstructure characterization of jujube cell

The detail of microstructure characterization testing was as followed: The collected pulp was crumbed by a sharp blade. Then, a thin layer of crumbed pulp which were putted on the slide and the other slips covered was observed with a Digital biological microscope (CX31RTSFJY) with a magnification of 400×.

### 2.4 Statistical analyses

A factorial design was used to study the effect of six different processing samples. Analysis of variance (ANOVA) with the Duncan test was carried out to verify whether there is a significant difference among different treatments in the investigated traits using SPSS 20.0 (SPSS Inc.) software. And *p* < .05 was considered to indicate statistical significance among different treatments.

## 3 RESULTS AND ANALYSIS

### 3.1 Juice yield

**FIGURE 1:**
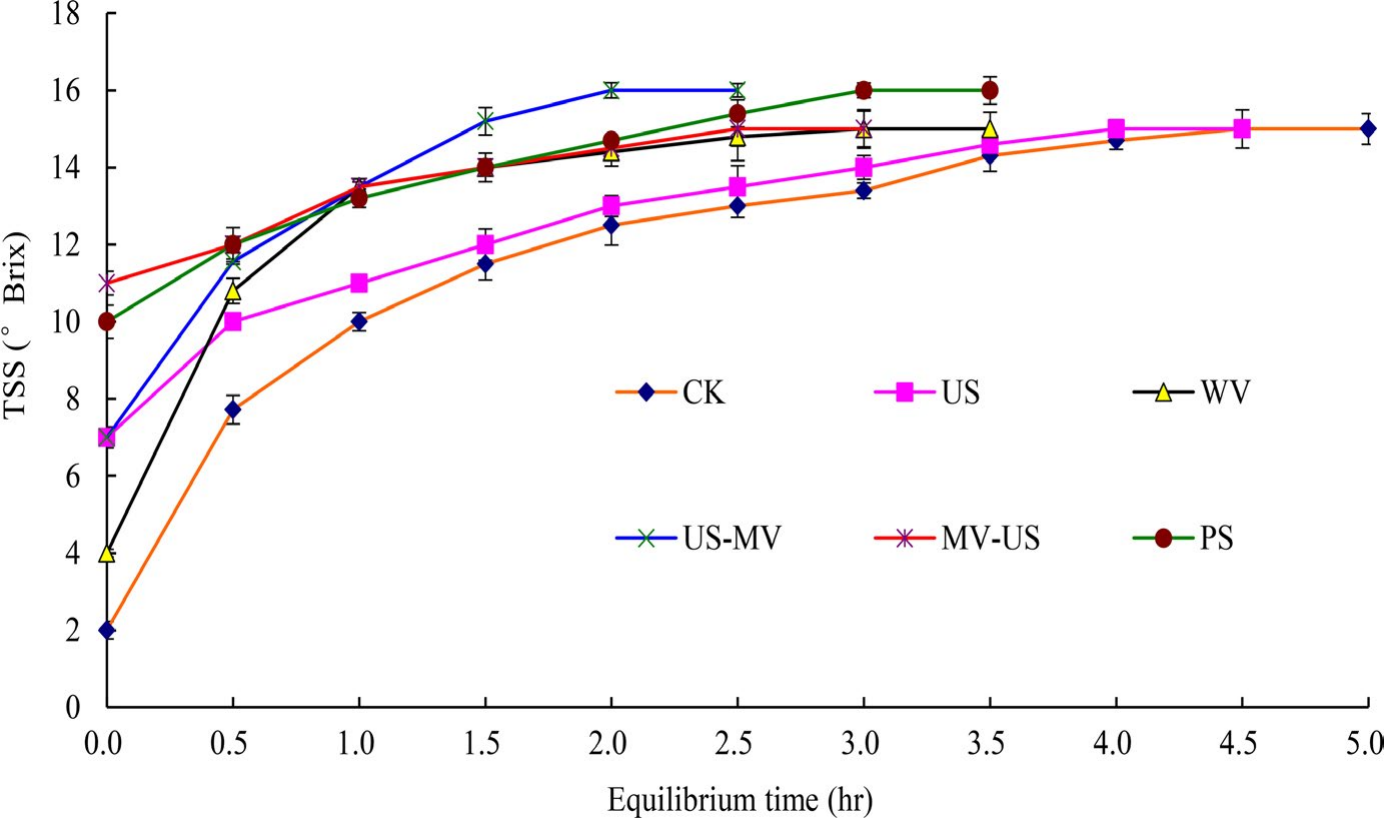
Effects of different treatments on the equilibrium time of TSS

The equilibrium time and juice yield were used as the index of production efficient. Figure 1 showed the equilibrium time of total soluble solids (TSS) after processing with different methods. The TSS of juice processed with US‐WV reached 16 °Brix quickly and steady. It took 2 hr to reach the equilibrium time. Additionally, the time of WV‐US was 3 hr and its highest point was 15 °Brix that was 6.67% lower than US‐WV. Compared to the latter, the time of the former reached the equilibrium in advance. Likewise, it had a time reduced about 1.2‐fold compared to the CK. According to the changes of the equilibrium time of the TSS, the effects of ultrasound and microwave in the independent form were revealed that the intensity of microwave was higher in comparison. No obvious difference was observed between ultrasound and microwave (*p* > .05). The equilibrium time was 3.5 hr and the TSS was 16 °Brix which processed by PS and it reached the top slowly. Thus, the synergetic form of ultrasound and microwave accelerated the yield of juice. Further, the juice acquired with synergetic form method was clearer than PS method.

### 3.2 The water‐soluble pectin

**FIGURE 2:**
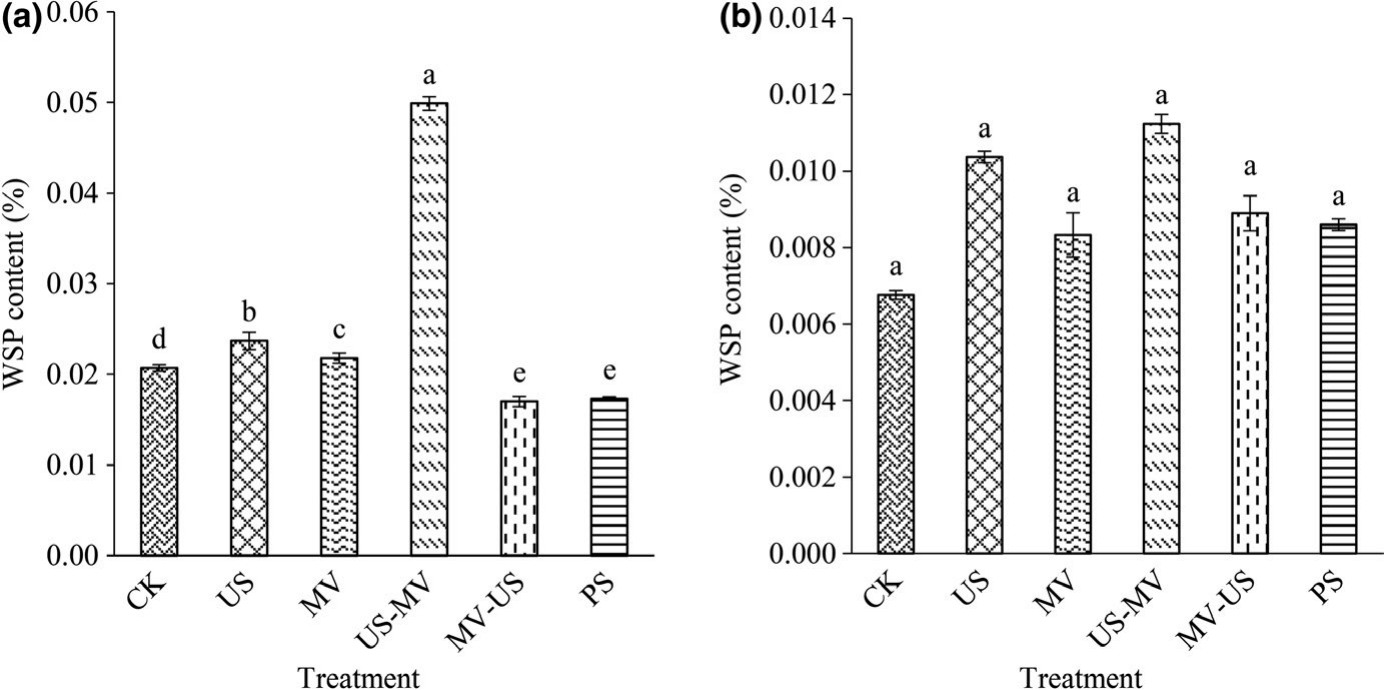
Effects of different treatments on water‐soluble pectin in fruit juice (a) and pulp (b)

When the cell wall was destroyed, the pectin of cell wall in which the liquid medium located was beneficial to the output of juice. The content of water‐soluble pectin (WSP) of jujube juice and pulp treated by diverse processing was shown in Figure 2. In all treated samples, the WSP of the juice treated with US‐MV was 0.0499%, showing the highest content (*p* < .05). It had higher 58.52% than the control (Figure 2a). Due to the different treatments, the treated pulp tissue showed a partly damaged cell structure (Figure 2b). The WSP of the pulp which treated by US‐MV in six treatments had no significant differences (*p* > .05). The possible reason for the tendency of variations might be due to the amount of WSP attached to the pulp was certain. The content of jujube juice and pulp in the other four groups was not high. Compared with other groups, ultrasonic and microwave treatment is more beneficial to jujube juice yield. The WSP of juice by US‐WV improved on the whole.

### 3.3 Protopectin

**FIGURE 3:**
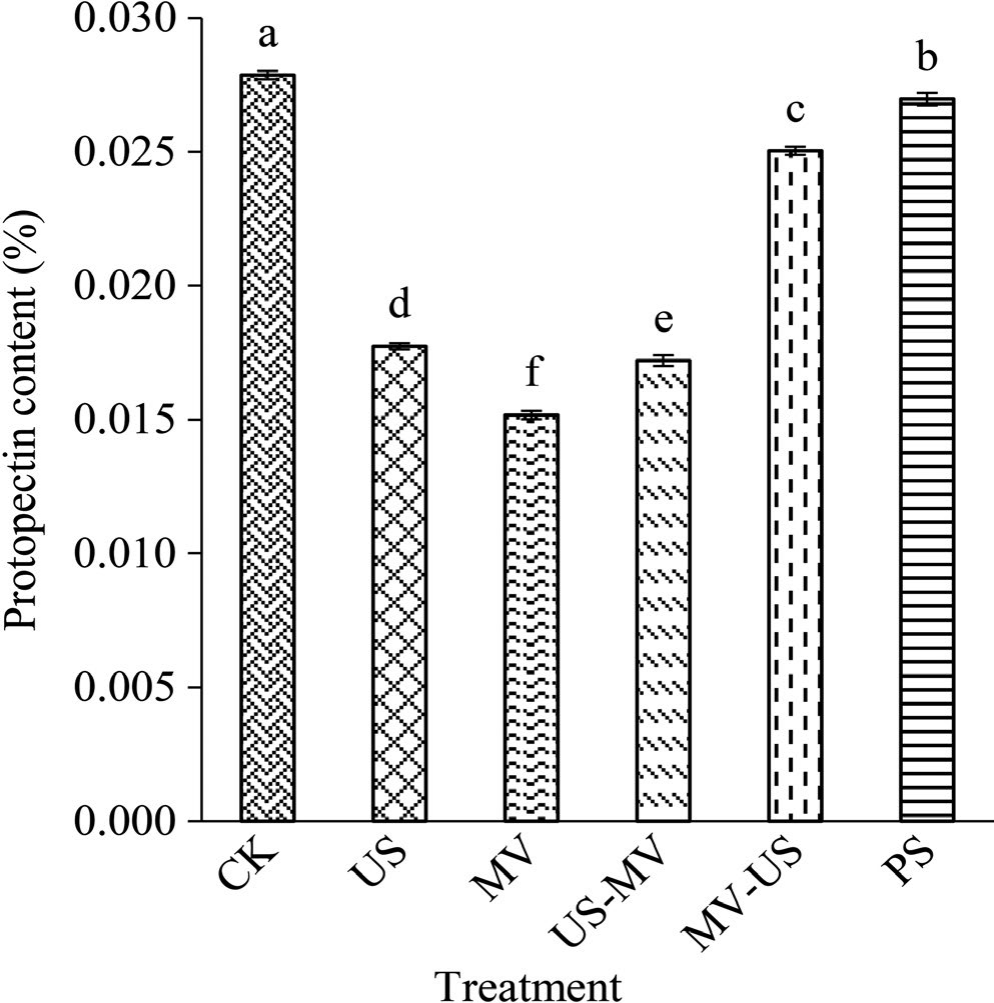
Effects of different treatment on protopectin in pulp

Protopectin as an insoluble substance is generally associated with plant polymers and cell debris having fiber‐like molecular structure in immature fruits. Therefore, protopectin presented only in jujube pulp. As Figure 3 showed, different processing had a significant effect on the content of protopectin. The protopectin content of US‐WV was significantly decreased and the loss ratio was 38.35% as compared to CK. The effects of ultrasound and microwave are decreased obviously both in the independent and the synergetic form at different conditions. Meanwhile, the pulp by pressing decreased about 3.23%. Among the treatments, microwave destroyed the protopectin in a large part. Due to the variation of sequence of ultrasound and microwave, the protopectin showed great discrepancy.

### 3.4 Galacturonic acid

**FIGURE 4:**
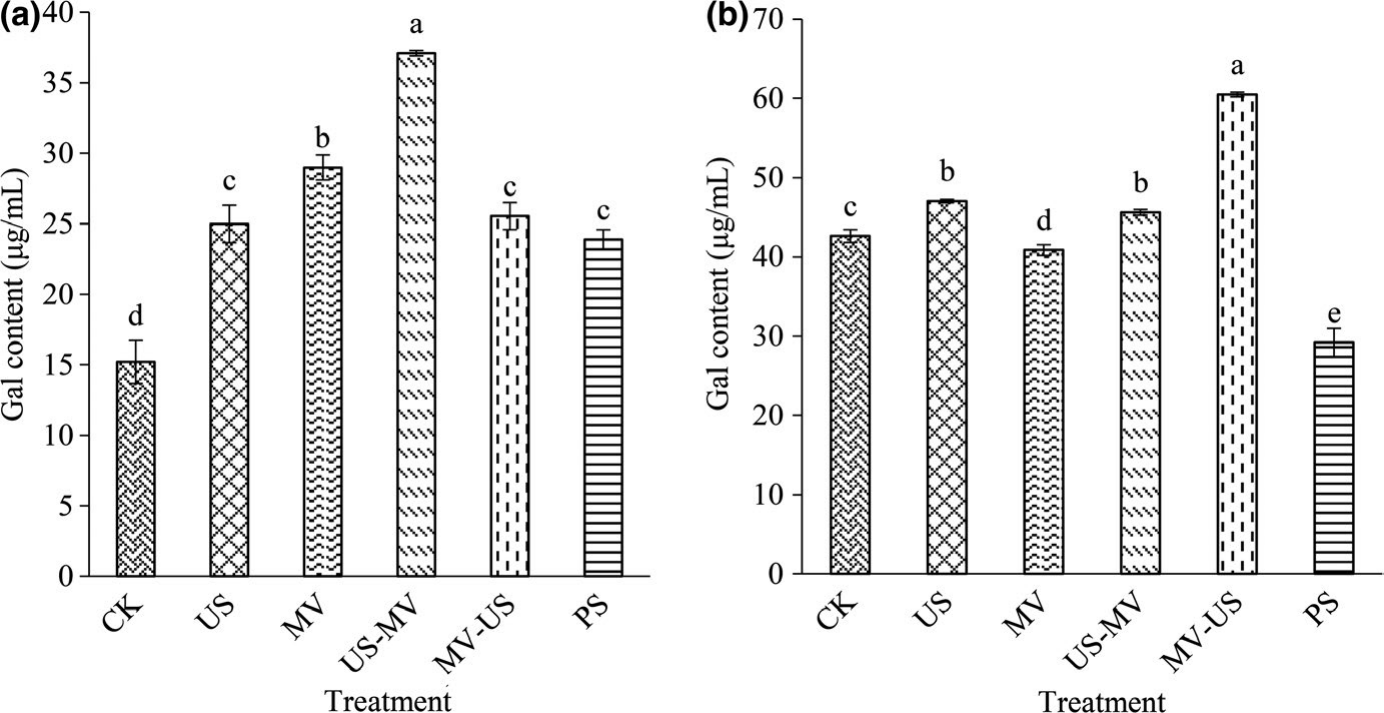
Effects of different treatments on the content of galacturonic acid in juice (a) and pulp (b)

Pectin is present in the cell wall and middle lamellae of fruit and vegetable. Galacturonic acid (GalA) is formed through degradation of the pectin structure (Rodsamran & Sothornvit, 2018). As was shown in Figure 4a, GalA content of the juice under the conditions of microwave and ultrasound was 60.84% and 52.44% higher than CK. By integrating microwave and ultrasound technology, the GalA content showed large variation. There were prominent differences between the two composite technologies. The US‐WV was 37.11 μg/ml, and the MV‐US was 25.56 μg/ml. The GalA variation of the pulp was exhibited in Figure 4b. When compared with each other, the result obtained with significant differences. The highest GalA content was in MV‐US, and the lowest was in PS. It demonstrated that the synergistic action of ultrasound and microwave would facilitate the hydrolysis of pectin.

### 3.5 Pectinase

**FIGURE 5:**
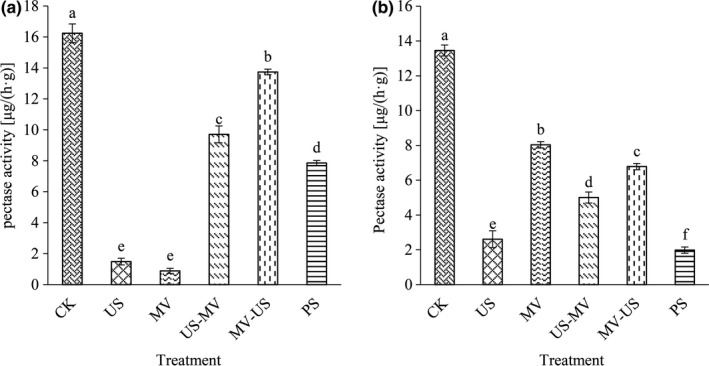
Effects of different treatment methods on pectinase activity in juice (a) and pulp (b)

Pectinases are a heterogeneous group of related enzymes of cell wall that hydrolyze pectin polysaccharides of plant tissues into small molecules and are mainly responsible for the variation in pectin properties. As a compound enzyme, pectinase is widely used for clarification of fruit juice (Rajdeo, Harini, Lavanya, & Fadnavis, 2016). Pectin was used as the basal material which digested to reflect the enzyme activity of juice and pulp during the extraction of jujube at the same time. And it was worth noted that microwave mainly passivated pectinase activity by heating, while ultrasound affected pectinase activity due to cavitation effect which caused the structure matrix to change (Arjmandi et al., 2016; Ma et al., 2016). The enzyme activity of juice of processing decreased significantly as Figure 5a showed. A half of the activity of control was that of pressing. Compared with the control, the enzyme activities of microwave and ultrasound were damaged tremendously by 37.25% and 50.41%. In addition, unexpectedly, we found the synaptic forms of microwave and ultrasound decreased a little in our study. Similar to the changes trend of the activity of pectinase of the juice, the activity of enzyme of pulp (Figure 5b) decreased with the treatments of microwave combination with ultrasound.

### 3.6 The total pectin

**FIGURE 6:**
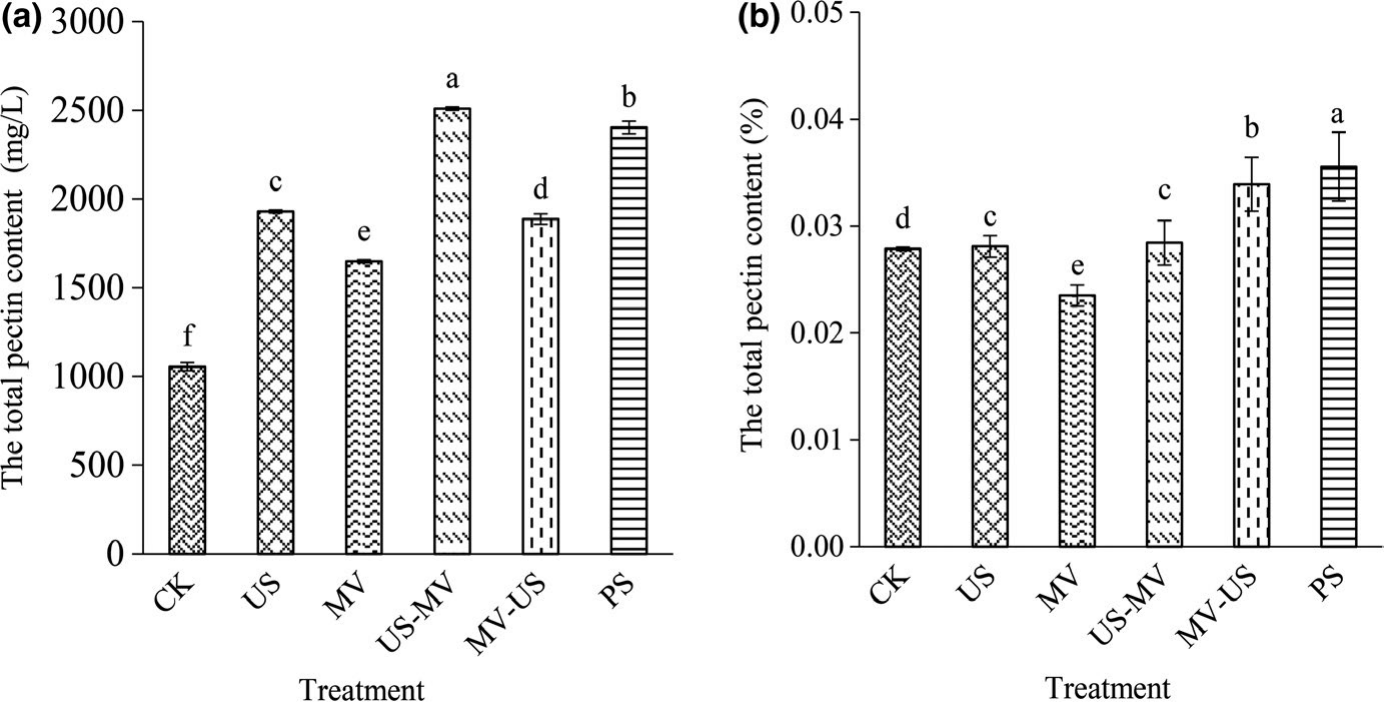
Effects of different treatment methods on total pectin content in juice (a) and pulp (b)

The total pectin content of juice was shown in Figure 6a. There was a significant increase in the total pectin content after processing (*p* < .05). In this test system, the total pectin content highest was in US‐WV (2,509.52 mg/L), and the second was in PS (2,402.85 mg/L). The pectin content in the other combined treatment (WV‐US) was only 1,872.62 mg/L, which was much lower (25.38%) than that in US‐WV. The total pectin content in pulp was calculated as a percentage (Figure 6b). The results indicated that there were significant differences between the control and other groups (*p* < .05). The content of pectin of pulp by pressing was the highest, followed by the microwave after ultrasonic treatment, which were 0.0356% and 0.0339%, respectively. Besides, the total pectin content of the microwave before ultrasonic was decreased by 16.22% when it compared to MV‐US.

### 3.7 Microstructure morphology of cell

**FIGURE 7:**
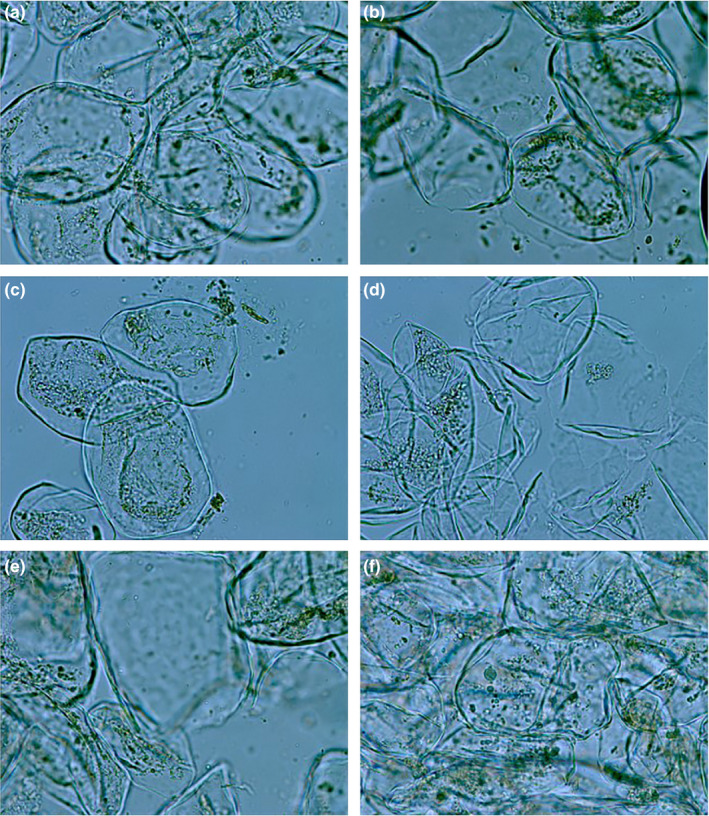
Effects of different treatments on microstructure (a) CK, (b) US, (c) MV, (d) US‐MV, (e) MV‐US, (f) PS

Figure 7 showed the digital biological microscope images of the treated and untreated groups. The surfaces of the treated underwent some morphological variations compared with the untreated control. The control check was regular and intact, whereas treated cells became shriveled and pitted, which led to the leakage of the contents of cell. The cell wall treated by MV‐US presented rupture and deformities, as well as the US‐MV (Figure 7d,e). Particularly, the cell walls of jujube treated by microwave after ultrasonic were damaged enormously. There was not intact cell wall almost. The leakage of the contents of cell was observed obviously in Figure 7c. The characteristics** **presented of cell wall demonstrated the microwave had the destroyed strength at some extents. Some cells turned from the normal round shape into irregular shape as Figure 7f showed. The structure of the cell wall became too nonrigid to maintain the cell morphology.

## 4 DISCUSSION

The effect of treatments on juice quality was determined by measuring the equilibrium time of total soluble solids (TSS), the content of pectin and galacturonic acid, and the activity of pectinase. The pectin is a heterogeneous complex polysaccharide consisting of mainly α‐(1–4)‐D‐galacturonic acid, including water‐soluble pectin and protopectin (Karnik, Jung, Hawking, & Wicker, 2016). Pectinase can degrade pectin, thus increasing fruit juice yield and reducing fruit juice viscosity. Although we confined subtle differences in the contents and component of cell wall from jujube juice and pulp, it is not difficult to figure out the mechanism of the interaction between them. In this study, those indices were found to be the major components of the cell wall by processing. As the cells were destroyed by treatments, the pectin substances were stripped from the cells, and the pectin was further decomposed into galacturonic acid as which the small molecules served. Those substances made the jujube juice had high yield and value. And the presence of pectinase facilitated the degradation of pectin. Owing to the degradations, it is beneficial to improve the filtration and clarification of juice.

Ultrasonic with cavitations’ effect can increase the intracellular energy of cell directly and accelerate the leakage of the contents of cell. It might result in pectin dissolve into small molecules, such as galacturonic acid. And the content of galacturonic acid increased reasonably. Microwave disrupts the structure of cell by heating. The fracted cell led to the separation of pectin from the cell wall at large extent. A previous study reported that the expansion with subsequent rupture of the cell walls which cause by microwave led to a better extraction of antioxidant compounds (Brenda et al., 2018). Similar to the trend of changes of ultrasonic, the pectin was hydrolyzed and pectinase was damaged when treated by microwave. Compared with natural extraction, both microwave and ultrasonic could prompt the soluble solid content of juice to reach the equilibrium faster and more stable. The content of pectin and galacturonic acid of juice which was treated by the microwave after ultrasonic was higher than other groups. The results might be due to the composite effect was enhanced, ultrasound destroyed the cellular structure mildly. Then with the existence of the thermal effect of microwave, the fracture strength was promoted and the production efficient of juice yield improved quickly. Another one of the composite group showed the destructiveness is smaller (Guo, Zhao, Li, Miao, & Zheng, 2019).

The micrograph of treated with different technologies showed further morphological alterations appeared in the cell wall. The changes in the cells might be due to the effect of the processing on the substance of cell wall, and then it resulted in the lysis of the cell wall, followed by the loss of intracellular materials. The processing of high voltage electrical discharges also could cause wall and cell membrane destruction (Homa & Somayeh, 2019; Xu et al., 2018). Consistently, pitted and ruptured cell walls were observed in each treatment. The ultrasonic after microwave method was more effective on exacerbating cell walls. Most of the treated cell walls became fragmentized, irregular, and shrunken. The intracellular substances without the constraint force of the cell walls aggregated together to be responsible for the reactions of liquid medium. The pectin peeled from cell walls also participated in as one of the members of the medium.

Microwave and ultrasonic might be used in juice prior to juice extraction in order to increase the extraction efficiency in the final product. In summary, microwaves in combination with ultrasound technologies presented an alternative in the processing of fruit juices with high yield, as demanded by consumers and industry. However, in‐depth researches on the compositional mechanisms, such as browning, and the application on jujube juices are still needed to be carried out.

## 5 CONCLUSION

Treating dried jujube with ultrasonic before microwave might improve the production efficient of dried jujube juice yield. The total soluble solids (TSS) was 16 °Brix, which was 6.67% higher than that of natural leaching. And its TSS reached equilibrium in 2 hr, which was faster than that of natural leaching, accelerating the ratio of the juice output at the same time. In summary, microwaves in combination with ultrasound technologies presented an alternative in the processing of fruit juices with high yield, which was demanded by juice industry.

## CONFLICT OF INTEREST

None.
